# Introduction to concept inventories for medical physics education

**DOI:** 10.1002/acm2.14130

**Published:** 2023-08-30

**Authors:** Ashley J. Cetnar, Abby Besemer, Victoria Bry, Courtney R. Buckey, Jay Burmeister, Anna Rodrigues, Leah Schubert, Michael Speidel, Steven Sutlief, Amy S. Yu

**Affiliations:** ^1^ Department of Radiation Oncology The Ohio State University Columbus Ohio USA; ^2^ Department of Radiation Oncology University of Wisconsin Madison Wisconsin USA; ^3^ Department of Radiation Oncology University of Washington Seattle Washington USA; ^4^ Department of Radiation Oncology Mayo Clinic Arizona Phoenix Arizona USA; ^5^ Department of Radiation Oncology Wayne State University Detroit Michigan USA; ^6^ Department of Radiation Oncology Duke University Durham North Carolina USA; ^7^ Department of Radiation Oncology University of Colorado Aurora Colorado USA; ^8^ Department of Medical Physics University of Wisconsin Madison Wisconsin USA; ^9^ Department of Radiation Oncology Banner MD Anderson Cancer Center Sun City Arizona USA; ^10^ Department of Radiation Oncology Stanford University Stanford California USA

**Keywords:** assessment, concept inventory, education

## Abstract

Concept inventories are multiple choice exams designed with the intention to test core concepts on specific subjects and evaluate common misconceptions. These tests serve as a useful tool in the classroom to assess value added by the instructor's educational methods and to better understand how students learn. They can provide educators with a method to evaluate their current teaching strategies and to make modifications that enhance student learning and ultimately elevate the quality of medical physics education. The use of concept inventories in introductory college physics courses revealed important gaps in conceptual understanding of physics by undergraduate students and motivated a shift of physics teaching towards more effective methods, such as active learning techniques. The goal of this review is to introduce medical physicists to concept inventories as educational evaluation tools and discuss potential applications to medical physics education by development through multi‐institutional collaboration.

## 1 REVIEW

The acquisition of knowledge and conceptual understanding during the course of medical physics graduate studies is fundamental to the success of students. Concepts are essential building blocks for student understanding and underlying development of scientific critical thinking. A common challenge that educators may face is whether students are comprehending the basic foundational concepts of the material or if the material is being memorized to be successful in the course. Educators in medical physics do not typically obtain formal training in education. Typical teaching experiences emulate apprentice relationships or on‐the‐job training and may not effectively or optimally prepare educators for teaching and assessing fundamental scientific concepts. Students who earn good grades in courses do not necessarily have a foundational understanding of the topic, so the reason for developing and implementing a concept inventory is to “probe the belief system, not intelligence.”^1^ A definition for concept inventories is “a multiple‐choice instrument designed to evaluate whether a person has an accurate and working knowledge of a concept or concepts.”^2^

Summative assessments are traditionally tests or quizzes that demonstrate a learner's understanding at the end of a lesson or course. These assessments are typically considered “high stakes” because of the relative importance of the evaluation to students. Formative assessments are low stakes, that is, not for a grade, evaluations in which the instructor is able to gain feedback about student learning throughout the course. Contrary to large‐scale testing, a type of summative assessment, where facts are assembled into an exam to test recall of a wide range of scientific areas, concept inventories are formative assessments that probe the fundamental understanding of the topic.^3^ The use of concept inventories as a low stakes (to the student) assessment provides valuable feedback to the instructor that allows instruction to be adapted to better communicate fundamental concepts.

Conceptual understanding refers to integrated comprehension of scientific knowledge and contextual application. The Force Concept Inventory, one of the first concept inventories used in introductory physics classes to assess students’ understanding of Newtonian concepts, started a revolution in how educators probe student conceptual understanding and misunderstanding that may seem trivial to the physics instructors.^1^ Poor student performance on this test encouraged the physics community to reconsider why students are not learning what instructors intend to teach. This idea of creating a test for conceptual understanding rapidly proliferated in the physics education community. The Force Concept Inventory has been adapted for various settings and concept inventories have been created for many different topics within physics education.^4^ The goal of this review is to introduce medical physicists to concept inventories as educational evaluation tools, and discuss potential applications to medical physics education.

### 1.1 Development of a concept inventory

**FIGURE 1:**
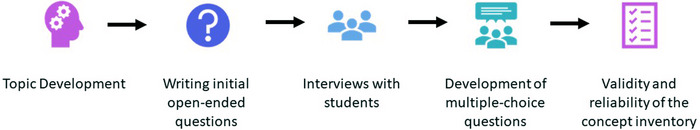
Steps in concept inventory development generated from microsoft powerpoint 16.

There are several ways to determine a student's conceptual understanding of a topic including conducting interviews or development of effective tests. Interviews can yield a rich and deep understanding of the way students perceive concepts, but unfortunately, these are time consuming for educators and researchers, so these approaches are challenging to scale. Effective tests, such as well‐designed concept inventories, can elicit a student's conceptual understanding and misconceptions; however, it must be created with this intention to be successful. Creating an effective and high‐quality concept inventory exam requires thoughtful development and validation. Figure 1 shows the primary steps in the process of concept inventory generation including topic development, writing initial open‐ended questions, interviews with students to identify common conceptions and misconceptions, development of multiple‐choice questions, and validity and reliability of the test.

### 1.2 Topic development

The first step in developing a concept inventory is selecting the core topic for the test. Adam and Weiman highlight the importance of selecting a subject matter of value to that educational community, and experts in the field should be surveyed to find out what is most important in the topic.^5^ Madsen, McKagen, and Sayre discuss two approaches, designing the test to be for either breadth or depth in a particular topic.^4^ Using the expertise of subject matter experts to determine important concepts, the areas of focus can then be defined more precisely within the context of education for defining the scope of the inventory.  Setting a clearly articulated goal for the evaluation is essential before creating specific items for the concept inventory.

Some resources available to medical physicists when considering the development of new concept inventories include textbooks, curricular recommendations for graduate schools and residencies, curricular requirements from accreditation bodies, and required topics for board certification. Review of emerging topics and assessment of the scope of particular topics can be expanded through literature review. Engaging with subject matter experts and review of literature can help establish content validity.

The original Force Concept Inventory test was limited to the topic of Newton's laws in effort to understand how balanced and unbalanced forces can lead to interesting static situations and dynamic phenomena. Without imposing such restrictions on the scope of content, a concept inventory test may fail to assess whether the student can extend a unified conceptual model to novel phenomena. One example of a conceptual model is that when the change in a physical quantity is proportional to the amount remaining, exponential behavior is obtained. This behavior is found in both nuclear decay and attenuation of primary radiation where the change in a physical quantity is proportional to the amount remaining. Another example is that radiation interactions in matter can be qualitatively understood by considering the mass and charge of the radiation. The extent to which such unifying principles consolidate a student's understanding of diverse phenomena can be quantified by a well‐developed concept inventory test.

#### 1.2.1 Development of open‐ended questions

**FIGURE 2:**
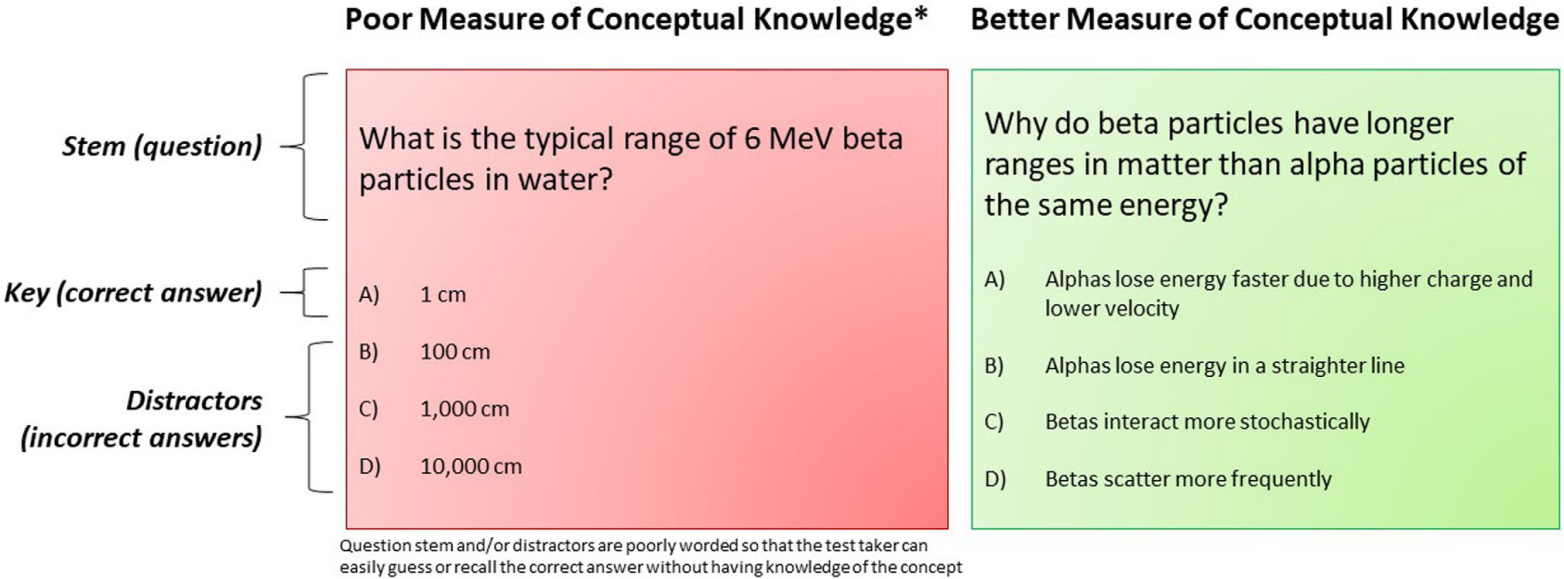
Process of creating a concept inventory through open‐ended questions, initial interviews, and creation of multiple‐choice questions.

After gaining the collective perspective from professionals in the field, open‐ended questions can be developed related to the fundamental concepts within the topic. These questions should be designed in such a way that they thoughtfully reflect the concepts to be tested, but do not have to be in the final form. Engelhardt suggests first writing stems in an open format and then administering them to a group of students within the targeted population to gain ideas and themes of novice student thinking.^6^, ^7^ The process of creating a concept inventory through open‐ended, initial interviews, and multiple choice questions is shown in Figure 2.

#### 1.2.2 Student interviews

Once the open‐ended questions are established, students should be interviewed in order to gain an understanding of their potential misconceptions.^5^ The interviews should be structured, not a conversation, providing opportunities for the students to say what they think without guiding them to respond a certain way.^5^ It is important not to aid the students during the questioning process by filling in the blanks in their understanding or leading them to think in a way that would not be representative of how the student would respond during a written examination. The interviewer cannot assume that students are providing the correct answers for the right reasons, so the interviewer should ask the students the reasoning for their answer.

#### 1.2.3 Multiple‐choice question development

**FIGURE 3:**
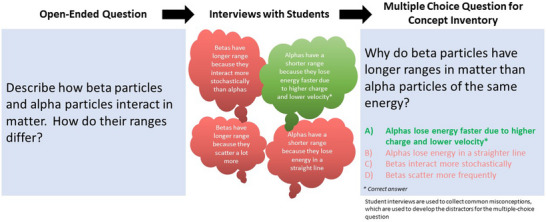
Example for stem, key, and distractors for poor quality and high‐quality questions.

Once student interviews are conducted, the collected information is used to convert the original open‐ended questions into multiple‐choice items. Multiple‐choice items can be separated into components^8^ such as the stem (the question) and answer choices consisting of a key (correct answer) and distractors (incorrect answers) shown in Figure 3. Linn and Gronlund provide several suggestions for developing these items including generating a stem that presents a definite problem, ensuring it is free of irrelevant material, and using items that use language familiar to the audience.^9^ A challenge in creating effective concept inventories is that some students are skilled at recognizing correct answers but do not have the ability to explain their reasoning for choosing it.^10^ Generating a set of plausible distractors based on student responses during interviews can help to negate this potential advantage. Specific misconceptions can be presented as plausible answers to form the distractors for each question.^6^

Once the range of student misconceptions has been shaped into multiple choice questions and reviewed by experts, another round of interviews should be conducted using the developed multiple‐choice questions. The second round of interviews ensure that the multiple‐choice questions are interpreted correctly. All of the distractors provided should be plausible solutions based on actual student misconceptions.

When developing questions for the concept test it is important to have an adequate number of multiple‐choice questions for the instructional objectives or fundamental concepts for the student learning. This will depend on the scope of the concept test, but Englehart recommends five items per objective for low stakes inventories but to start with more than 20 questions so that the best can be selected to remain for the final version.^6^  As an additional metric to the accuracy of student responses, Madsen, McKagan, & Sayre required students to accompany their answers to the questions with a confidence scale for a concept test in quantum mechanics, since students typically have very little prior knowledge about the subject.^4^

### 1.3 Concept inventory validity and reliability

While there are many strategies for assessment, creating an exam that accurately reflects the effects of specific teaching methods on student learning can be a difficult feat. Unfortunately, in the field of physics education research, few concept tests have been validated by statistical methods.^2^ Having small class sizes in medical physics programs makes it difficult for a researcher to validate a new instrument. It is important that quantitative validation of the concept inventory be performed prior to using it for educational research. We propose the development of multi‐institutional research collaborations to accomplish the accrual for larger numbers of student responses.^11^ Depending on the purpose of the study, two different methods (among others described in the literature) for validation include classical test theory or item response theory.^6^, ^12^

Within classical test theory, it is assumed the score on the assessment is reflective of the concept of interest where the assessment can be evaluated using several metrics including difficulty, discrimination, validity, and reliability.^7^ Difficulty is a measure of how often a particular question is answered correctly. Discrimination is a measure of whether the question is being answered correctly by students performing well on the overall test. For example, if a question is missed by many of the high scoring students, it is important to investigate why, including evaluating the language of the question stem and the language and quality of the distractors. Validity is the measure of whether a test is measuring what it intends to assess, and reliability is the measurement of how stable the metric is when repeated. A limitation of this approach includes the assumption that the relationship between the scores on the assessment are linear to the fundamental understanding of the student. Many references are available in the literature for details calculating these metrics.^13^, ^14^, ^15^

Item response theory is a non‐linear model based on the correlations between a student's response on an individual test item and the student's understanding of the material. Rasch measurement is an example of a specific type of assessment within item response theory which considers the result of the exam as a series of probabilistic results based on the student's interactions with the questions.^16^ Unlike classical test theory, its underlying assumption is that the test is independent of both item difficulty and student ability, but can be challenging to implement due to its complexity and large number of respondents required for building accurate models. It is important that quantitative validation of the concept inventory is performed prior to its use for education research, but the methods for validation could use classical test theory or item response theory depending on the purpose of the study.

Concept inventories are typically validated for a specific student population (e.g., undergraduate students, graduate students). Careful consideration should be taken when using concept inventories for a student population that is, different than the one which the inventory was validated.  It is important that the inventory continues to be tested and evaluated by the research community in order to maintain its applicability. After validation of the initial concept inventory, further optimization of the concept inventory can be investigated to reduce the number of questions while still obtaining meaningful results. As an example, the Colorado Learning Attitudes about Science Survey (CLASS) survey instrument was optimized to reduce the overall number of questions with improved psychometric properties, when compared to the original.^17^

### 1.4 Application of concept inventories in the classroom

A well‐developed concept inventory is primarily used to measure the effectiveness of the instructor's educational methods and determine whether instruction resulted in a gain in student understanding. By giving a pre‐test and post‐test during the semester, the instructor can calculate the raw gain and the adjusted gain.^18^ This assessment is not to grade individual student performance, but to understand the change in understanding of the class as a whole as a reflection of instruction. When administering the concept inventories, the environment for dissemination of the test should be standardized since the timing of the test and the external motivation for completing the formative assessments have been shown to influence consistency of student responses.^19^

Some assessments are considered criterion‐referenced such that a predetermined standard is set for expected performance. While each test is different, in Hestenes, Wells, and Swackhamer's paper evaluating the Force Concept Inventory,^1^ they defined a score of 80% on the Force Concept Inventory as a Newtonian thinker and 60% as a baseline for sufficient Newtonian understanding. However, the educational research community does not recommend using concept inventories for determination of “cut scores” without validation against an adequate standard. In the initiation of concept inventories in the field of medical physics, the primary goal is to validate a concept inventory so that educators can have standardized assessment instrument to critically reflect upon their teaching instead of defining levels of student expertise in the subject matter.

### 1.5 Concept inventories in medical physics

Students enter the field of medical physics with many ideas about radiation derived from popular media, news, and personal medical experiences. Concept inventories can be developed in fundamental medical physics topics to help uncover misconceptions that might hold the student back. Creators of the original Force Concept Inventory, used to test misconceptions in Newtonian mechanics, cited the finding that “commonsense beliefs about motion and force are incompatible with Newtonian concepts in most respects.”^1^ They identified six concepts: kinematics, each of Newton's three laws, superposition, and the different kinds of forces as the focus of their concept inventory. To uncover student misunderstandings, each Force Concept Inventory test item is a forced choice between these concepts and commonsense alternatives.

Medical physics has its own core concepts, for which an absence of understanding leads the student to rely on rote memorization and calculation formalisms that lack coherent meaning. We believe the study of medical physics conceptual understanding will be an area of future education research interest within our field as educators are looking to best teach our students and residents.

## 2 DISCUSSION

Concept inventories can be an invaluable tool for medical physics instructors. As an educator, it is essential to evaluate and assess your students’ understanding of core concepts. Often, students can score well on exams even if they do not completely understand the core concepts. Concept inventories not only provide a helpful way to gauge the student's understanding of the course material but also allow instructors to identify the gaps in their teaching methods. Since concept inventories are developed and created by content experts, they can help instructors design their course in a way that ensures these basic concepts and core ideas are well‐covered.  Additionally, because the distractors within the concept inventory multiple‐choice items are based upon misconceptions held by real students, the instructor can prospectively identify and discuss those potential pitfalls through focused class activities and homework assignments.

Another advantage of concept inventories is that the exam taken at the beginning of the course can be used to gauge the students’ current level of understanding. The course material could then even be personalized for each group of students based on the results of the initial concept inventory that year. Moreover, concept inventories can help instructors better understand the students’ knowledge at the conclusion of the course, strengthening the curriculum for the future. Concept inventories also provide a way for instructors from different institutions to measure the common misconceptions of their students with a concept inventory. Therefore, with the use of concept inventories in a longitudinal fashion, we can use the data to have a broader program‐wide curricular discussion across the nation to provide a better medical physics education to our students.

As in other physics disciplines, concept inventories can be useful in identifying non‐intuitive concepts and common misconceptions in medical physics that students may bring with them into medical physics graduate study.  The use of concept inventories has also demonstrated the capability of identifying teaching methods, such as active learning techniques, which help overcome weaknesses and misconceptions in student understanding.  Significant efforts have been made to develop the relevant content^20^ and to identify the most successful teaching strategies with the American Association of Physicists in Medicine.  The development and deployment of concept inventories would help instructors both identify important impediments in the teaching of these concepts and provide a tool to evaluate changes in teaching strategies.

Once appropriately validated, concept inventories are tools that can be used by any educator in the medical physics field to evaluate conceptual understanding of core medical physics concepts.  This is a first for the medical physics education field, in which a common tool can be used to measure conceptual understanding.  This tool provides educators with a method to evaluate their teaching strategies to enhance student learning and ultimately elevate the quality of medical physics education.

### 2.1 Disclaimer

The authors strongly advise against using concept inventories to determine a grade for a course (or section of a course) or be used as a final exam. The concept inventory questions, answers, or student performance results should not be returned to the student whenever possible since concept inventories have had time and resources invested in the validation of the instrument as a research tool which would be compromised with wide dissemination. It is not advised that the results be used as an evaluation of individual students but should be used as a tool for the self‐reflection of educators, continuous improvement of educational methods, and further research. Authors believe that developed concept inventories should be used as low stakes evaluation tools, for example, not intended to impact the student's status in a course such as for official grades. Concept inventories are validated for a specific student population (e.g., undergraduate students, graduate students). Careful consideration should be taken when using concept inventories for a student population that is, different than the population for which the inventory was validated.

## AUTHOR CONTRIBUTIONS

**Ashley J. Cetnar**: Conceptualization; methodology; writing—original draft; development of concept inventories and reviewing manuscript. **Abby Besemer**: Development of concept inventories; writing and reviewing manuscript. **Victoria Bry**: Development of concept inventories; writing and reviewing manuscript. **Courtney R. Buckey**: Development of concept inventories; writing and reviewing manuscript. **Jay Burmeister**: Development of concept inventories; writing and reviewing manuscript. **Anna Rodrigues**: Writing—review and editing. **Leah Schubert**: Development of concept inventories; writing and reviewing manuscript. **Michael Speidel**: Writing and reviewing manuscript. **Steven Sutlief**: Development of concept inventories; writing and reviewing manuscript. **Amy S. Yu**: Development of concept inventories; writing and reviewing manuscript.

## CONFLICT OF INTEREST STATEMENT

There are no conflicts of interest from the authors.
